# Local Mutational Pressures in Genomes of Zaire Ebolavirus and Marburg Virus

**DOI:** 10.1155/2015/678587

**Published:** 2015-12-20

**Authors:** Vladislav Victorovich Khrustalev, Eugene Victorovich Barkovsky, Tatyana Aleksandrovna Khrustaleva

**Affiliations:** ^1^Department of General Chemistry, Belarusian State Medical University, Dzerzinskogo 83, 220116 Minsk, Belarus; ^2^Laboratory of Cellular Technologies, Institute of Physiology of the National Academy of Sciences of Belarus, Academicheskaya 28, 220072 Minsk, Belarus

## Abstract

Heterogeneities in nucleotide content distribution along the length of Zaire ebolavirus and Marburg virus genomes have been analyzed. Results showed that there is asymmetric mutational A-pressure in the majority of Zaire ebolavirus genes; there is mutational AC-pressure in the coding region of the matrix protein VP40, probably, caused by its high expression at the end of the infection process; there is also AC-pressure in the 3′-part of the nucleoprotein (NP) coding gene associated with low amount of secondary structure formed by the 3′-part of its mRNA; in the middle of the glycoprotein (GP) coding gene that kind of mutational bias is linked with the high amount of secondary structure formed by the corresponding fragment of RNA negative (−) strand; there is relatively symmetric mutational AU-pressure in the polymerase (Pol) coding gene caused by its low expression level. In Marburg virus all genes, including C-rich fragment of GP coding region, demonstrate asymmetric mutational A-bias, while the last gene (Pol) demonstrates more symmetric mutational AU-pressure. The hypothesis of a newly synthesized RNA negative (−) strand shielding by complementary fragments of mRNAs has been described in this work: shielded fragments of RNA negative (−) strand should be better protected from oxidative damage and prone to ADAR-editing.

## 1. Introduction

Viruses from Filoviridae family are known as causative agents of dangerous hemorrhagic fevers. The most known virus from that family is Zaire ebolavirus which recently (in 2014-2015) caused a new outbreak [1, 2]. The genome of a Filoviridae virus is built from a relatively long (about 19000 nucleotides) single RNA negative (−) strand [2].

Separate mRNAs are synthesized on a matrix of a single RNA negative (−) strand, while there is just one site for RNA-dependent RNA-polymerase binding situated at the 3′-terminus of a genome [3]. There are conserved nucleotide motifs near the boarders of open reading frames: transcription start signals at which polymerase should start a new mRNA molecule and transcription stop signals (polyA sequences) at which polymerase should finish a separate mRNA molecule [3]. Soon after the infection a so-called gradient of transcription can be observed: each next gene is transcribed at lower rates than the previous one [4, 5]. This phenomenon was thought to be caused by dissociation of the RNA-polymerase complex with RNA negative (−) strand at the transcription start/stop signal [3] or by the sliding of nontranscribing polymerases along the genome [6]. The process of stop/start signals bypassing works normally closer to the end of the viral life cycle when RNA negative (−) strand serves as a template for full-length complementary RNA positive (+) strands synthesis [2]. Then full-length RNA positive (+) strands serve as templates for full-length daughter RNA negative (−) genomes production [2].

Transcriptional gradient may be one of the factors leading to differences in mutational pressure directions or intensity along the length of a genome [7]. That is why genomes of viruses from Filoviridae family are of a great interest for researchers working on the mutational pressure theory [8–10]. To find out how exactly transcriptional gradient influences mutational pressure we studied nucleotide content distribution along the genomes, estimated nucleotide mutations direction, and analyzed secondary structure content in separate parts of RNA positive (+) and negative (−) strands.

Accumulation of certain nucleotide mutations is associated with specific phases of the viral life cycle [11]. The data on the general mutational pattern and deviations from it can shed some light on specific features of viral transcription, replication, and latency. Moreover, estimation of mutational pressure direction may help to choose the best (the less mutable) proteins or their parts (epitopes) for recombinant [12] or synthetic vaccine creation [13].

There are such components of nonspecific intracellular antiviral defense as adenosine deaminases able to bind just double-stranded fragments of RNA (ADAR superfamily [14]) and cytosine deaminases able to bind only single-stranded fragments of RNA (APOBEC1 family [15]) or DNA (APOBEC3 family [16]). ADAR-editing leads to A to G or A to T mutations (due to formation of inosine from adenine) on the respective RNA strand [17]. APOBEC1-editing leads to C to U mutations [15]. Genomes of Zaire ebolavirus and Marburg virus were shown to be edited by ADAR [4, 18–20]. However, it is unknown whether A to G transitions have higher rates of occurrence relative to other types of mutations in all the coding regions of Zaire ebolavirus or just in some of their fragments. Theoretically, the ability of each RNA fragment to form double-stranded structures (stems of hairpins) should determine whether this fragment is prone to ADAR-editing or to APOBEC1-editing. Double-stranded fragments of both DNA and RNA are known to be protected from oxidative damage (mostly causing cytosine deamination and guanine oxidation) better than single-stranded ones [21]. From this point of view the amount of secondary structure may be one of the main determinants of mutation rates and direction in a given fragment of RNA. On the other hand, mutational pressure may influence RNA secondary structure formation (in case of G-pressure) or destruction (in case of A-pressure) [22].

Biases in mutation occurrence rates lead to biases in nucleotide usage [8, 10]. However, this process of the nucleotide usage change usually takes a long time [23] even in fast evolving viruses. That is why it is important to use the method for mutational pressure direction estimation which avoids consideration of nucleotide usage biases. Some fragments of viral RNA with biased (relative to the rest of the genome) nucleotide usage may be just traces of insertion events that are slowly moving towards the common nucleotide usage [9]. Other fragments of RNA may demonstrate their own local mutational pressure because of such factors as autonomous transcription [11] or hidden transcription stop signals [24, 25]. In this work we found out which of those fragments in Zaire ebolavirus and Marburg virus genomes are “active volcanoes,” and also we provided evidences that the cause of their local mutational pressure existence should not be associated with autonomous transcription.

## 2. Materials and Methods

In this study we used all available nucleotide sequences of* Zaire ebolavirus* and* Marburg virus* from GenBank. Nucleotide sequences of coding regions from reference genomic sequences (NC_002549 and DQ447653.1, resp.) have been used in the BLAST (http://blast.ncbi.nlm.nih.gov/Blast.cgi) search algorithm. BLAST search has been performed only among the sequences of the corresponding species:* Zaire ebolavirus* (taxid:186538) and* Marburg virus* (taxid:11269). Those sets of sequences have been downloaded and processed. Only unique full-length sequences have been left in each alignment: repeated 100% identical sequences, as well as partial sequences, have been deleted. Resulting sets of sequences can be found in the DATAZE.xlsx and DATAMV.xlsx files from Supplementary Material available online at http://dx.doi.org/10.1155/2015/678587.

We used the information on the deep sequencing experiments performed by Shabman et al. [4] available in Supplementary Material Table  S2 to their article, as well as in NCBI BioProjects under accession numbers PRJNA258131 (for* Zaire ebolavirus*) and PRJNA264121 (for* Marburg virus*). Total RNA in the abovementioned experiment was isolated by using Trizol (Invitrogen) from Vero cells (African green monkey kidney epithelial cells) or differentiated Thp1 cells (human monocytic leukemia cells) infected with EBOV Mayinga (CDC isolate number 808011) or MARV-Ang (CDC isolate number 200501379) at multiplicity of infection of 3 [4]. RNA molecules containing poly-A tails were purified with Invitrogen Dynal oligo dT magnetic beads and then RNA-Seq libraries have been created using NEB Next mRNA-Seq kit using 18 cycles of PCR [4]. Obtained Illumina HiSeq 100 nt reads were mapped to the reference Zaire ebolavirus and Marburg virus sequences mentioned above by using the TopHat/Cufflinks software suite v2 with the default settings [4].

For each of the unique full-length sequences of coding regions from Zaire ebolavirus and Marburg virus secondary structure has been predicted by the CentroidFold algorithm [22]. That algorithm is able to predict secondary structure for sequences up to 2000 nucleotides in length. Because of this reason Pol coding regions have been cut into 4 parts (1650, 1650, 1650, and 1689 nt in length for Zaire ebolavirus; 1740, 1740, 1740, and 1749 nt for Marburg ebolavirus). The minor nucleoprotein VP30 (the viral protein with the molecular mass of 30 KDa) coding region of Marburg virus have been cut into two parts (192 and 654 nt) according to the results of the deep sequencing experiment [4]. NP coding region of Zaire ebolavirus has been cut into two parts (900 and 1320 nt), while GP coding regions of both viruses have been cut into three parts according to the positions of cross-folds in uracil and cytosine usages (987, 663, and 378 nt for Zaire ebolavirus GP; 651, 393, and 963 nt for Marburg virus). NP coding region of the Marburg virus has also been cut into two parts (843 and 1244 nt) according to the boarders determined for its homologue from Zaire ebolavirus genome. Each of the abovementioned sequences has been converted to its reversed complement with the help of the MEGA 6.0 program [26]. Secondary structures have been predicted for reversed complement sequences by the CentroidFold algorithm [22].

Alignments of sequences for each coding region have been studied with the “VVTAK VarInvar” algorithm (http://chemres.bsmu.by). The algorithm calculates nucleotide usage in invariable fourfold and twofold degenerated sites from third codon positions and compares it with average nucleotide usage in all fourfold degenerated and twofold degenerated sites which have no nonsynonymous mutations in the whole alignment (stable sites). If the usage of a nucleotide is higher in invariable sites than in stable sites, we consider this nucleotide to be nonmutable and state that its usage is growing. If the usage of a nucleotide is lower in invariable sites than in stable sites, we consider this nucleotide to be mutable and state that its usage is decreasing [11].

Graphs representing nucleotide usage distribution along the length of a genome have been built with the help of MS Excel. We used reference genomes of* Sudan ebolavirus* (NC_006432),* Tai Forest ebolavirus* (NC_014372),* Bundibugyo ebolavirus* (NC_014373),* Reston ebolavirus* (NC_004161),* Lloviu cuevavirus* (NC_016144), Vesicular stomatitis Alagoas virus Indiana 3 (NC_025353.1), and Chimpanzee adenovirus type 3 (HC469240.1). To test the significance of the differences in secondary structure amounts for different sequences we used Mann-Whitney* U* test (the distribution of that variable is not normal according to the Shapiro-Wilk test).

## 3. Results

### 3.1. Nucleotide Content Distribution along the Lengths of Viral Genomes from Filoviridae Family

**Figure 1:**
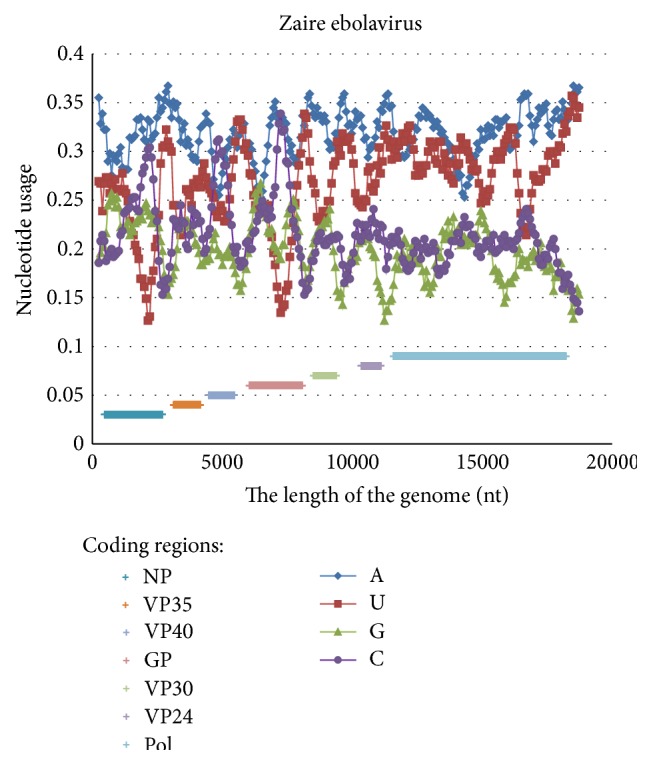
Nucleotide content distribution along the length of the reference Zaire ebolavirus genome. Sliding window size is 490 nucleotides and the step is 70 nucleotides.

Along most of the length of the Zaire ebolavirus reference genome the usage of adenine (31.87 ± 0.30%) is significantly higher than the usages of other nucleotides (see Figure 1). The average usage of uracil in sliding windows 490 nucleotides in length each is equal to 26.89 ± 0.53%. In general, the usage of cytosine is higher than the usage of guanine in the studied viral genome (21.39 ± 0.42% versus 19.85 ± 0.42%, *P* < 0.001).

Usages of adenine and guanine do not exhibit significant variations along the whole Zaire ebolavirus genome. In contrast, the level of cytosine demonstrates three sharp peaks (see Figure 1). The first peak of cytosine usage reaches 30.48% at the nucleotide #2205. This peak corresponds to the variable 3′-part of the first gene encoding nucleoprotein (NP) [27]. The second peak of cytosine usage has a maximum of 31.22% and corresponds to the whole coding region of the matrix protein (VP40). The third peak is the highest one (33.88% of cytosine) and it is situated in the area encoding highly variable (mucin-like) part of the outer glycoprotein (GP) [27].

Interestingly, the first and the third peaks of cytosine usage are associated with the lowest levels of uracil usage (12.65% and 13.47%, resp.). However, the second peak of cytosine usage is not associated with low level of uracil (see Figure 1).

According to the mutational pressure theory [8], there should be a general mutational AU-pressure in the genome of Zaire ebolavirus that is asymmetrically biased towards adenine, while in three regions of that genome there are local deviations from that direction. To find out the causes of those deviations we first built similar graphs for other viruses from the same genus and family.

**Figure 2:**
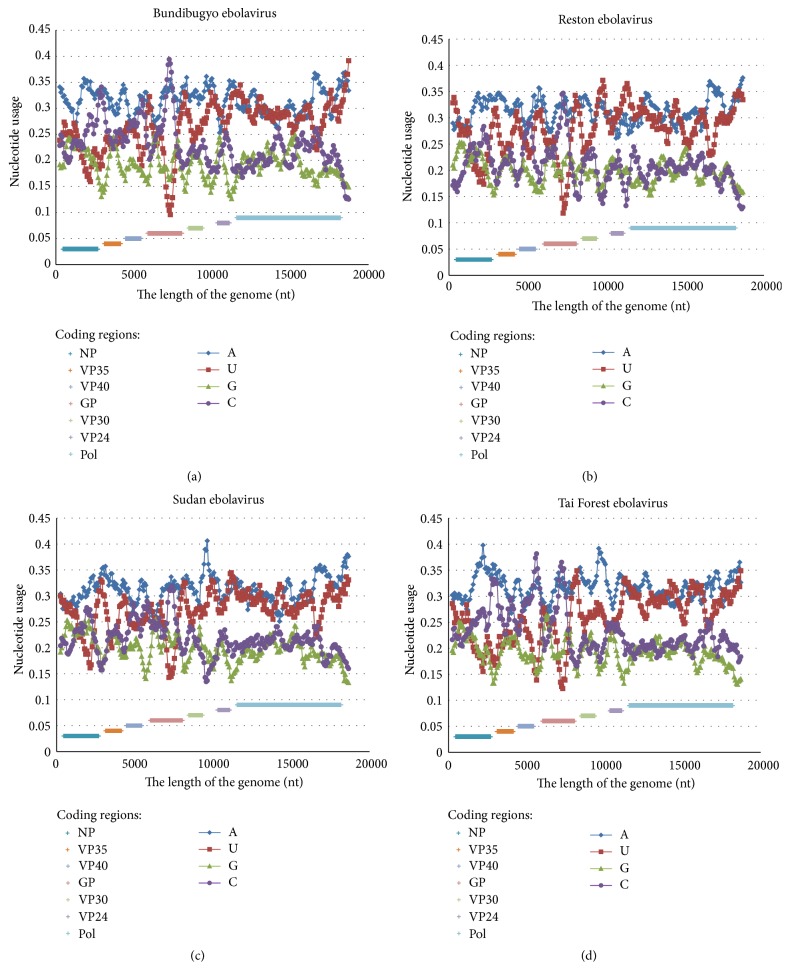
Nucleotide content distribution along the length of the reference genomes of Bundibugyo ebolavirus (a), Reston ebolavirus (b), Sudan ebolavirus (c), and Tai Forest ebolavirus (d). Sliding window size is 490 nucleotides and the step is 70 nucleotides.

Genomes of other ebolavirus species (*Sudan ebolavirus*,* Tai Forest ebolavirus*,* Reston ebolavirus,* and* Bundibugyo ebolavirus*) have patterns of nucleotide content distribution similar to that for Zaire ebolavirus genome (see Figure 1). However, there are some variations in height of the three cytosine content peaks described above. For the Bundibugyo ebolavirus genome maximal cytosine usage levels are 34.08%; 31.63%; and 39.39% (Figure 2(a)). For the Reston ebolavirus genome those three cytosine usage peaks are lower: 28.37%; 28.98%; and 34.49% (Figure 2(b)). They are relatively low in the genome of Sudan ebolavirus as well: 27.14%; 28.98%; and 31.43% (Figure 2(c)). However, in the Tai Forest ebolavirus genome cytosine peaks are sharp and high: 33.27%; 38.16%; and 36.53% (Figure 2(d)). According to these data, some ebolavirus species may still evolve towards higher cytosine content in the three parts of their genomes, while others are drifting to homogenous nucleotide content distribution.

**Figure 3:**
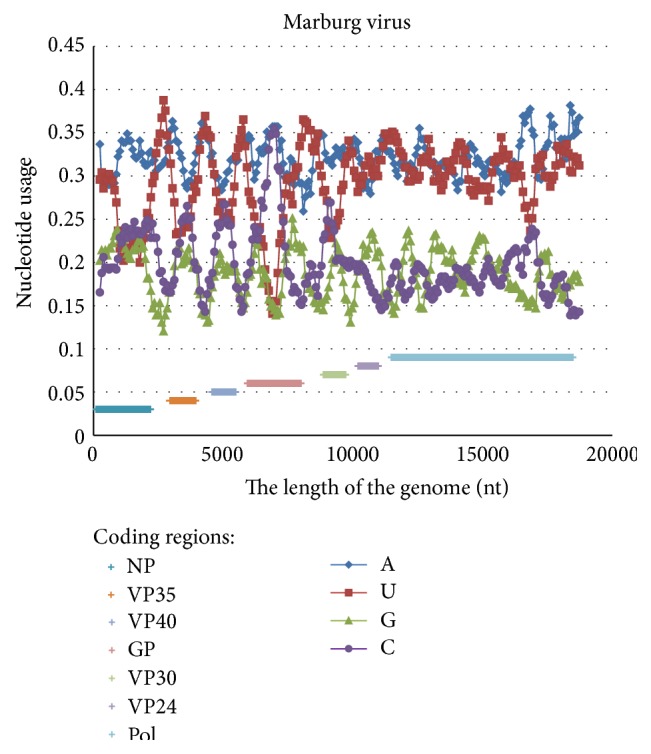
Nucleotide content distribution along the length of the reference Marburg virus genome. Sliding window size is 490 nucleotides and the step is 70 nucleotides.

In the genome of Marburg virus (the one from the same Filoviridae family) one can observe just a single cytosine content peak associated with the lowest uracil usage (see Figure 3). That single cytosine usage peak is situated in the middle part of the GP coding region. Cytosine usage is, in general, higher in NP, VP35 (polymerase complex protein), VP40, GP, and VP30 coding regions than in the noncoding regions between them (Figure 3), but it is never higher than 27%. These data provide us with an evidence that the middle part of GP coding region contains some features making it cytosine-rich in genomes from both* Ebolavirus* and* Marburgvirus* genuses.

**Figure 4:**
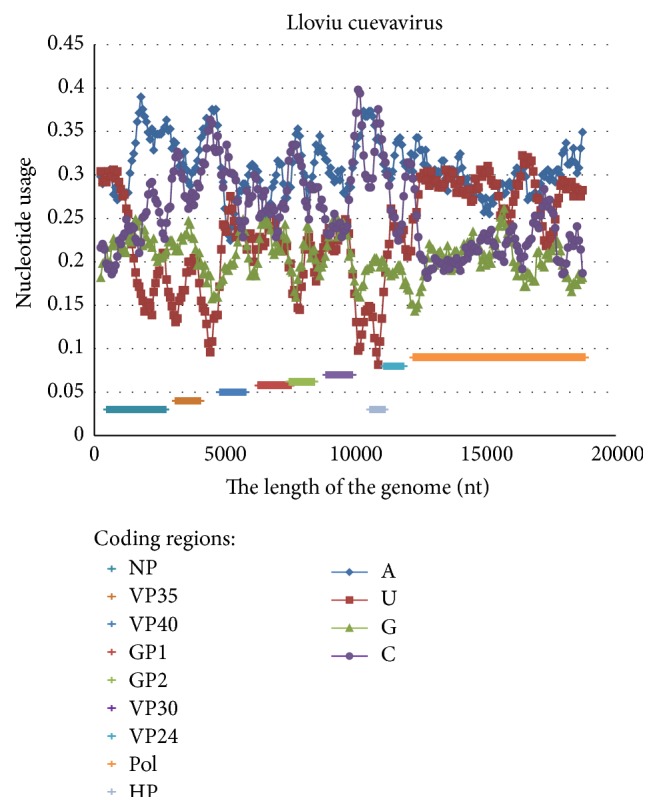
Nucleotide content distribution along the length of the reference* Lloviu cuevavirus* genome. Sliding window size is 490 nucleotides and the step is 70 nucleotides.

There is a third genus of the Filoviridae family represented by* Lloviu cuevavirus* [28]. In its genome one can observe two separate patterns of nucleotide content distribution (Figure 4). In the 5′ two-thirds of the genome the usages of adenine and cytosine are higher than the usages of uracil and guanine (one may suggest the existence of mutational AC-pressure). In the 3′ third of the genome represented by the RNA-dependent RNA-polymerase coding region the usages of adenine and uracil are higher than the usages of guanine and cytosine (one may suggest the existence of mutational AU-pressure). High difference between cytosine and uracil usages especially can be observed in NP, VP35, VP30, and VP24 (membrane-associated protein) coding genes, while in VP40 and GP coding genes that difference is not as high.

Because of some reasons fragments of Filoviridae RNA positive (+) strand may be either under AU-pressure or under AC-pressure (and under general A-pressure). There is a hypothesis of transcription-associated mutational pressure [29] that may be used to explain this heterogeneity of pyrimidines usage in viral genomes. C-rich (or U-rich) genes or fragments of genes may be transcribed at higher (or lower) rates than other fragments and accumulate different types of nucleotide mutations because of this reason. However, there are no data on autonomously expressed RNAs from Filoviridae genomes. Fortunately, there are results of the deep sequencing experiments for Zaire ebolavirus and Marburg virus published in 2014 [4].

### 3.2. The Direct Dependence between the Nucleotide Coverage in the Deep Sequencing Experiments and Cytosine Usage for Zaire Ebolavirus and Marburg Virus

**Figure 5:**
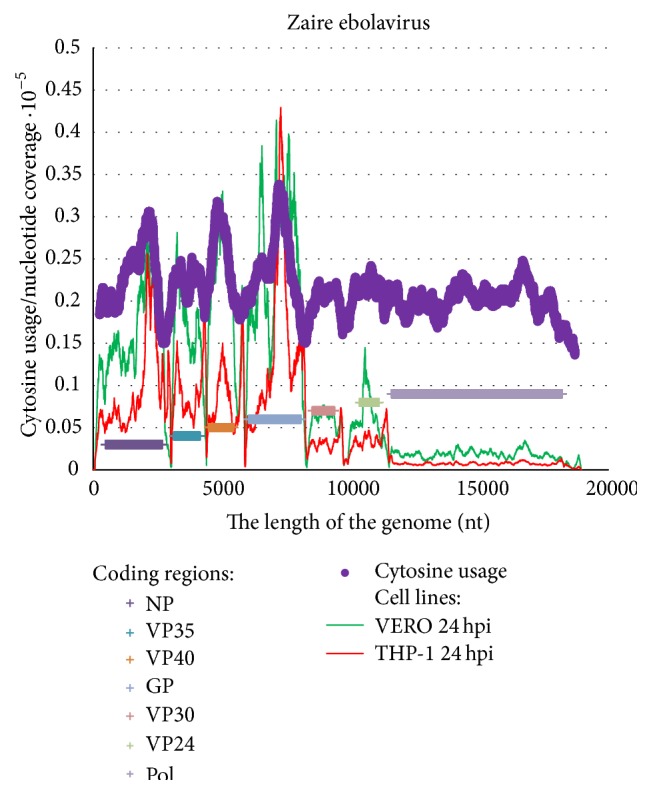
Cytosine content distribution along the length of the reference Zaire ebolavirus genome. Sliding window size is 500 nucleotides and the step is 1 nucleotide. Nucleotide coverage in deep sequencing experiments in VERO and THP-1 cells 24 hours after infection [4] is shown.

**Figure 6:**
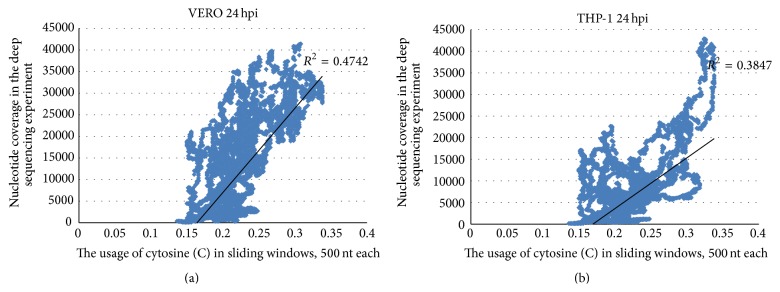
Correlation between cytosine usage in fragments of Zaire ebolavirus reference genome and nucleotide coverage in VERO (a) and THP-1 (b) cells 24 hours after infection.

In Figure 5 we show cytosine usage in sliding windows of 500 nucleotides (with a step equal to one nucleotide) and nucleotide coverage (multiplied by 10^−5^ to make graphs comparable with each other) for Zaire ebolavirus. The deep sequencing has been performed in two cell lines [4]. We used the information provided by the authors of that study [4] as a supplementary material to their own article [4]. There is a direct correlation between nucleotide coverage of mRNAs in both VERO (*R* is equal to 0.6886) and THP-1 (*R* = 0.6202) cell lines with the cytosine content of the sequence (Figure 6), while the total amount of viral mRNAs (especially of the abundant fragments) is higher in VERO cell line [4]. There is also indirect correlation between nucleotide coverage and uracil usage (*R* is −0.6841 for VERO cells and −0.6513 for THP-1 cells). Guanine and adenine usages show much weaker dependence on nucleotide coverage (coefficients of correlation are 0.5069 and −0.3134 for VERO cells, 0.2644 and −0.0008 for THP-1 cells). For C-rich fragments of NP and GP genes there are corresponding peaks of nucleotide coverage in both VERO and THP-1 cell lines (but in VERO cells those peaks are wider), while VP40 mRNA (or its fragments) is not as abundant in THP-1 cells 24 hours after infection as in VERO cells.

High cytosine usage of the VP40 coding region may be explained by the high expression level of its mRNA in the late period of the infection happening in certain types of cells with weak nonspecific antiviral defense [4]. This high expression may be linked to the lack of characteristic secondary structure of the transcription start signal of the VP40 gene. Indeed, all other transcription start signals form a structure of a single relatively long (9–15 base pairs) hairpin [5] or two short hairpins (Table 1), while in case of VP40 there is just a single short (3 base pairs) hairpin. Probably, RNA-polymerase or another transcription factor recognizes a linear nucleotide motif and starts transcription effectively for all the signals, but the transcription factor which inhibits transcription in the late period of infection recognizes linear nucleotide motif better if that motif has a certain secondary structure.

**Figure 7:**
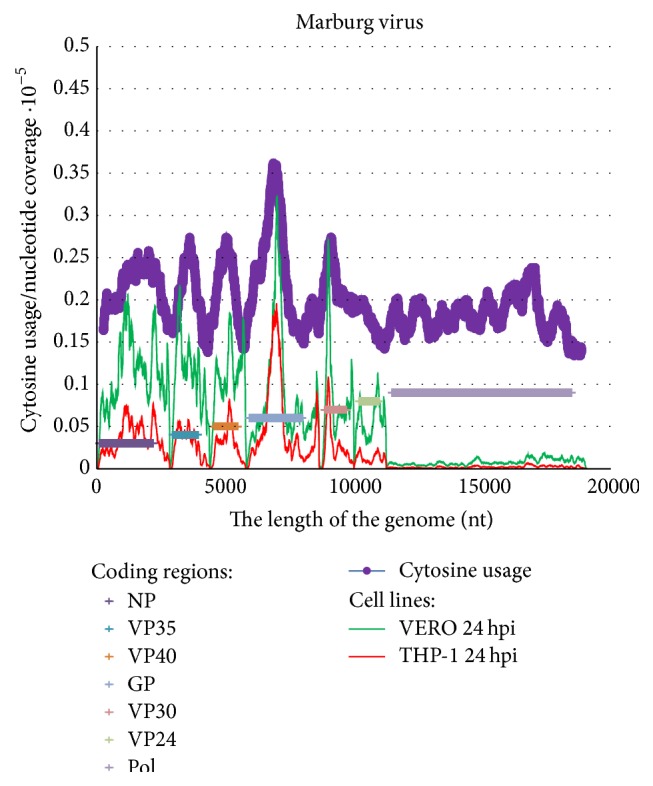
Cytosine content distribution along the length of the reference Marburg virus genome. Sliding window size is 500 nucleotides and the step is 1 nucleotide. Nucleotide coverage in deep sequencing experiments in VERO and THP-1 cells 24 hours after infection [4] is shown.

**Figure 8:**
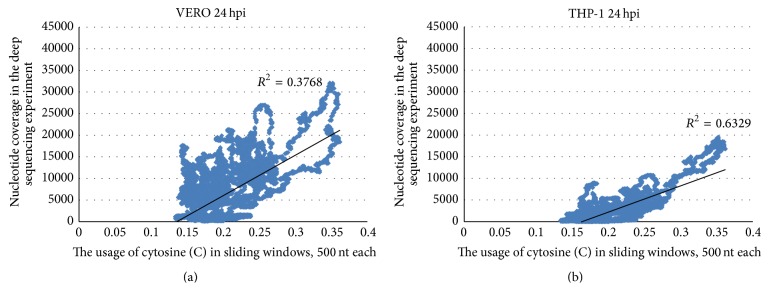
Correlation between cytosine usage in fragments of Marburg virus reference genome and nucleotide coverage in VERO (a) and THP-1 (b) cells 24 hours after infection.

In Figure 7 one can see that the highest peak of nucleotide coverage for Marburg virus (in the middle of GP coding region) is really associated with the highest peak of cytosine usage. However, the number of relatively C-rich mRNA fragments and the number of highly abundant mRNAs (both in VERO and THP-1 cell lines) are lower for Marburg virus than for Zaire ebolavirus. Because of this reason there is a strong correlation between those two parameters (see Figure 8). The coefficient of correlation between nucleotide coverage and cytosine usage is 0.6138 for VERO and 0.7956 for THP-1 cells. For uracil usage there is inversed correlation: the coefficient of correlation is −0.5689 in VERO cells and −0.7279 in THP-1 cells. Usages of other nucleotides are not mutually connected with nucleotide coverage according to the following coefficients of correlation: −0.0200 and −0.0678 for guanine and 0.0877 and 0.1453 for adenine in two corresponding cell lines. The VP40 coding region of the Marburg virus shows no deviations from the transcription gradient [4], as well as other genes (except small fragments of GP and VP30).

The nucleotide coverage shows that certain fragments of mRNAs are more abundant than others. High abundance of an mRNA fragment may be the consequence of its high expression or of its resistance to degradation by numerous cellular RNAses [30]. There is a general belief that single-stranded fragments of RNA are degraded faster than double-stranded ones [31]. So, we tested the hypothesis that high abundance of NP and GP mRNA fragments may be explained by the higher amount of secondary structure elements in them. Unexpectedly, results showed that those fragments are able to form lower number of hairpins than others.

### 3.3. Amount of Secondary Structure Is Lower for Highly Covered Fragments of mRNAs

Analysis of secondary structure amount (the percentage of nucleotides which make base pairs) showed that RNA fragments highly covered in the deep sequencing experiment [4] demonstrate lower tendency to form hairpins.

In Table 2 one can find medians for the percentages of nucleotides forming hydrogen bonds for Zaire ebolavirus coding regions and their fragments. Highly covered part 2 of the NP coding region has much lower (*P* < 0.001) amount of nucleotides forming “stems” (33.33%) than part 1 of the same coding region (42.00%) demonstrating decreased nucleotide coverage. The middle part of the GP coding region has lower amount of nucleotides forming “stems” than part 1 and part 2 of the same gene. However, the difference between part 1 and part 2 of GP is not very high (35.26% versus 34.69%, *P* < 0.001), but it is significant (Table 2). For 9 out of 87 sequences of the GP part 2 the amount of nucleotides in “stems” is lower than 30%, while there are no such poorly structured sequences of the GP part 1.

Similar situation can be found in the Marburg virus genome (Table 3). There are two peaks of nucleotide coverage in that genome situated in the part 2 of the GP coding region and in part 1 of the VP30 coding region. Both of them demonstrate extremely low percentage of paired nucleotides (20.36% and 6.77%). To explain these facts one has to review the principle of the deep sequencing. Only polyadenylated mRNAs have been used for this experiment [4]. So, differences in nucleotide coverage inside the same coding region cannot be explained by the autonomous expression of short RNAs. All mRNAs have been randomly sliced by RNAses. Then those fragments have been sequenced using the random set of primers. The lower the amount of “stems” for a given short fragment of mRNA is, the better the binding of random primers with it should be. The low amount of nucleotides which have already formed hydrogen bonds to the ones from the same RNA molecule increases the rate of successful hydrogen bond formation between that poorly structured fragment of RNA and the primer with antisense sequence.

The process taking place during deep sequencing experiment may be partially repeated* in vivo*. Viral mRNAs should be sliced by cellular RNAses into numerous relatively short fragments [30]. Those fragments of the positive (+) strand should have a high chance to interact with full-length negative (−) strands of the viral genome. The lower the amount of secondary structure in the fragment of viral mRNA, the higher the chance that it will bind the complementary fragment of the newly synthesized RNA negative (−) strand. Shielded fragments of viral RNA negative (−) strands should be protected from APOBEC1-editing and should be prone to ADAR-editing. As a result, the usage of cytosine will be increasing in certain fragments of viral RNA positive (+) strand.

To test this hypothesis one should compare amounts of stems in fragments of RNA negative (−) strand. For Zaire ebolavirus NP part 1 and part 2 as well as Marburg ebolavirus GP fragments amounts of stems in negative (−) strand are very close to each other (Tables 2 and 3). For Marburg virus VP30 coding region the amount of stems is much lower in part 1 than in part 2 for both positive (+) and negative (−) strands. It means that for those three fragments of viral genes (Zaire ebolavirus NP part 2, Marburg virus GP part 2, and Marburg virus VP30 part 1) the possibility of RNA duplex formation (by fragments of mRNAs and antisense part of genomic RNA negative (−) strand) is really high.

The middle fragment of Zaire ebolavirus GP coding region (GP part 2) should be highly covered only in the reference strain (in which it really has low percentage of stems equal to 27.75%, unlike GP part 1 with 38.70% and GP part 3 with 52.91%) but not in many other strains. Moreover, fragments of GP mRNA corresponding to its part 2 will rarely bind antisense part of the negative (−) strand because of the high amount of stems made by it (49.77%). To clarify the causes of nucleotide content distribution heterogeneities in Zaire ebolavirus and Marburg virus genomes we analyzed current directions of nucleotide mutations in corresponding coding regions and their fragments of interest.

### 3.4. Mutational Pressures in Viral Genes and Their Fragments

The test performed by the “VVTAK VarInvar” algorithm shows which nucleotides are mutable in a given coding region or its fragment. If we assume that in regions with elevated cytosine usage mutations leading to the occurrence of C have higher frequency than mutations leading to its disappearance, the usage of cytosine in invariable fourfold degenerated sites must be significantly higher than its usage in stable fourfold degenerated sites [11]. As one can see in Table 4, the usage of cytosine in fourfold degenerated sites (C4f) is indeed significantly higher in invariable sites than in stable sites of NP part 2, VP40, and GP part 3. It means that there is ongoing mutational C-pressure in those three fragments of Zaire ebolavirus RNA positive (+) strand, unlike in all other coding regions and their parts.

There is also an interesting trend in uracil mutability that can be observed in Table 4. In three last coding regions (VP30, VP24, and Pol) uracil usage is increasing, while in other coding regions (except just VP35) uracil usage is decreasing. This finding is in agreement with the fact that fragments with low nucleotide coverage are enriched by uracil. Three last coding regions demonstrate low expression rates and so fragments of those mRNAs should not cover negative (−) strand as frequent as fragments of other mRNAs.

Adenine usage is increasing throughout the whole RNA positive (+) strand of Zaire ebolavirus. It means that there is a permanent mutational A-pressure, most likely caused by cytosine deamination and guanine oxidation on the negative (−) RNA strand leading to G to A and C to A mutations on the positive (+) RNA strand.

Guanine usage in fourfold degenerated sites is decreasing in all coding regions and their fragments in Zaire ebolavirus genome, except NP part 1, GP part 1, and VP30. Those parts of positive (+) strand with nonmutable guanine residues are situated near C-rich fragments. Probably, they form double-stranded fragments together during the time of the full-length positive (+) strand existence. Because of this reason abovementioned fragments may accumulate more A to G transitions and less G to U transversions than other ones.

Guanine usage in twofold degenerated sites is growing only in NP part 2 and GP part 2 (Table 5). This fact can also be explained by the ADAR-editing of RNA positive (+) strand formed secondary structure with nearby situated coding regions or by the lower rates of C to U transitions in RNA negative (−) strand covered by fragments of mRNAs.

Unexpectedly, cytosine usage in twofold degenerated sites is decreasing in NP part 2 and in GP part 2 providing evidence that overall cytosine usage in these parts of positive (+) strand is growing due to the higher ratio between A to C and C to A transversions more likely associated with RNA negative (−) strand shielding than with RNA-editing.

Adenine and uracil usages in twofold degenerated sites of Zaire ebolavirus coding regions show variable behavior. It is important to highlight that pure mutational AU-pressure (when both A and U are decreasing in both fourfold and twofold degenerated sites) has been found only in Pol coding region.

In Marburg virus coding regions and even in their C-rich parts the usage of cytosine in fourfold degenerated sites is decreasing according to the results of the “VVTAK VarInvar” algorithm (Table 6). The usage of guanine is also decreasing in fourfold degenerated sites throughout the whole genome, while the usage of adenine is growing. As for the usage of uracil, it is growing in two last coding regions of the Marburg virus genome (VP24 and Pol), in part 1 of GP and in part 2 of VP30 coding region. Probably, the usage of cytosine in GP part 2 and VP30 part 1 is decreasing slower than in other parts of the positive (+) strand, while in general it is going down. It means that there are some additional factors stimulating local C-pressure in Zaire ebolavirus in contrast to Marburg virus.

In twofold degenerated sites of Marburg virus coding regions adenine usage is always growing and guanine usage is decreasing, while uracil usage behaves in variable manner (Table 7). Cytosine usage in twofold degenerated sites is growing in VP30 coding region only.

### 3.5. The Hypothesis of the Negative (−) RNA Strand Shielding by Complementary Fragments of mRNAs

The mechanism of cytosine-rich fragments creation and disappearance in genomes of Filoviridae viruses can be described by the following model. There is overall mutational A-pressure in viral genomes caused by C to U and G to U mutations on the negative (−) RNA strand. Adenine usage is growing mostly in fourfold and twofold degenerated sites due to the negative selection eliminating amino acid mutations [2]. However, some parts of proteins are under a weak negative selection [27]. In fragments of coding regions encoding variable parts of proteins (such as middle mucin-like part of GP) adenine usage has been growing not just in third but also in first and second codon positions. It is known that A-rich sequences of RNA usually have very small amount of secondary structure [22]. So, mutational A-pressure has led to the formation of mostly unstructured mRNA fragments. Such unstructured fragments of mRNA bind complementary negative (−) strand better than structured fragments. Because of this reason certain parts of negative (−) RNA strand should usually be covered by fragments of mRNAs, while other parts should not be “shielded.” Parts of RNA negative (−) strand covered by fragments of mRNAs are protected from guanine oxidation: the rates of C to A transversions on the complementary positive (+) strand should be decreased in such fragments. Moreover, double-stranded regions should be edited by ADAR: the rates of T to C transitions and T to A transversions should be higher in fragments of RNA (+) strand which are complementary to highly “shielded” fragments of RNA negative (−) strand. As a result, the usage of C is starting to grow in such unstructured A-rich parts of positive (+) RNA strand. The mild increase in cytosine level does not cause significant increase in the amount of secondary structure. The growth of C is possible only in case sufficient amount of viral mRNA fragments survives until the time of RNA negative (−) strand synthesis. As one can see in Figures 5 and 7, this factor depends on the cell type: in VERO cells the number of mRNAs is much higher for both viruses than in THP-1 cells. The growth of C usage on the positive (+) strand fragment is associated with the growth of G usage on the complementary negative (−) strand fragment. When the usage of G reaches a certain threshold level, the fragment of negative (−) strand starts to form more hairpins: G is prone to form not only G:C but also noncanonical G:U pairs [22]. Cytosine usage on RNA positive (+) strand fragment will continue its growth because the corresponding part of RNA negative (−) strand is already self-shielded. In such conditions the fragment of positive (+) strand may even acquire more hairpins than before and it will not stop the growth of cytosine inside it.

From this point of view, three peaks of cytosine usage found in Zaire ebolavirus genome can be described as “active volcanoes” with increasing cytosine content, while the single cytosine usage peak in Marburg virus genome is similar to the “sleeping volcano” which can be awaken by any factor increasing the amount of GP mRNA in cells infected by that virus.

## 4. Discussion

Viral genome may accumulate nucleotide mutations in different periods of the life cycle. Below we are going to discuss our results in light of known information about mutational processes. First of all, viruses with RNA negative (−) genomes should accumulate mutations when they are starting replication process (in the beginning of the infection) and when newly synthesized daughter genomes are packing into virions. In both conditions genomes of negative (−) polarity should be single-stranded. However, those single-stranded genomes should form secondary structure elements. The higher the amount of stems in a given part of the RNA negative (−) strand, the higher the rate of its ADAR-editing leading to A to G transitions occurrence and the lower the rate of its APOBEC1-editing, the lower the rate of its contacts with oxidative agents [16]. The concrete example of highly structured fragment of RNA negative (−) strand is the fragment complementary to the Zaire ebolavirus GP part 2 that demonstrates ongoing C-pressure.

In this study we are faced with the facts which can be interpreted as evidence of a new mechanism of asymmetric mutational pressure existence. Fragments of mRNAs may bind antisense parts of RNA negative (−) strands at the final steps of viral replication (when RNA negative (−) strands are synthesized, while the amount of partially degraded previously transcribed mRNAs is still high). This binding will lead to double-stranded RNA fragments formation. The outcome of this event will be the same as in the case with extensive secondary structure formation by a fragment of RNA negative (−) strand: the usage of C in the corresponding fragment of RNA positive (+) strand will grow (or will decrease more slowly than in other fragments). There are several factors influencing the success of this RNA negative (−) strand shielding by fragments of mRNAs. First of all, there must be sufficient level of gene transcription to produce enough mRNA fragments. Indeed, the last gene of both Zaire ebolavirus and Marburg virus (Pol) demonstrates very low expression level. This factor affects the nucleotide content of Pol gene: the usage of U is high (and it is keep growing), while the usage of C is low. Unshielded RNA negative (−) strand should show lower rates of ADAR-editing and higher rates of guanine oxidation (both factors lead to cytosine usage decrease and uracil usage increase in corresponding fragments of RNA positive (+) strand).

In contrast, highly expressed VP40 gene shows elevated usage of C and ongoing C-pressure. However, this gene is highly expressed only in Zaire ebolavirus, just in VERO cells and closer to the end of the infection process (after 24 hours and not even after 12 hours [4]). So, changes in gene expression associated with the cell type (namely, with the presence or absence of specific inductors or repressors) may influence mutational pressure direction. It is very likely that all coding regions of* Lloviu cuevavirus* (Figure 3), except Pol, have been highly expressed and accumulated high amount of cytosine due to the shielding effect. It is important to mention that Filoviridae viruses are usually able to survive in cells of different hosts (at least, in fruit bats and in primates) [2, 28]. Probably, directions of mutational pressure for some parts of their genomes are different when they infect different hosts.

Low amount of secondary structure elements makes the chance of successful RNA hybridization with its antisense molecule higher. This fact has been confirmed in this study. Fragments of genes with lower amount of secondary structure demonstrate elevated cytosine usage relative to fragments of same genes with higher amount of hairpins. This observation correlates well with the above written hypothesis of RNA negative (−) strand shielding, but it does not correlate with two other hypothetical mechanisms of asymmetric mutational pressure. Genomic positive (+) strand of RNA may become a target for oxidative damage during transcription (when it forms single-stranded transcriptional bubbles [7]) and during replication (after its own synthesis and before the synthesis of daughter RNA negative (−) strands). The lower the amount of secondary structure elements made by a fragment of that RNA positive (+) strand is, the lower the usage of C should be (due to cytosine deamination). So, there are two opposite effects of the low secondary structure amount in the fragment of single-stranded RNA negative (−) virus gene. The “shielding” effect will prevail if the expression level of a corresponding mRNA is relatively high and when the process of alien RNA degradation is somehow inhibited. A good example of the successful RNA negative (−) strand “shielding” may be the situation with Zaire ebolavirus NP part 2. In Marburg virus GP part 2 and VP30 part 1 RNA negative (−) strand “shielding” effect (that should exist according to the results of the deep sequencing experiments [4]) is not enough to prevent cytosine loss, but it, probably, makes that process slower.

The information on mutational pressure directions in different coding regions of Zaire ebolavirus is necessary for the creation of viral vector vaccines. Ideally, mutational pressure directions in the viral vector should be the same as in genes from Zaire ebolavirus. Otherwise, significant part of resulting proteins will have amino acid substitutions making vaccine antigens different from natural ones.

**Figure 9:**
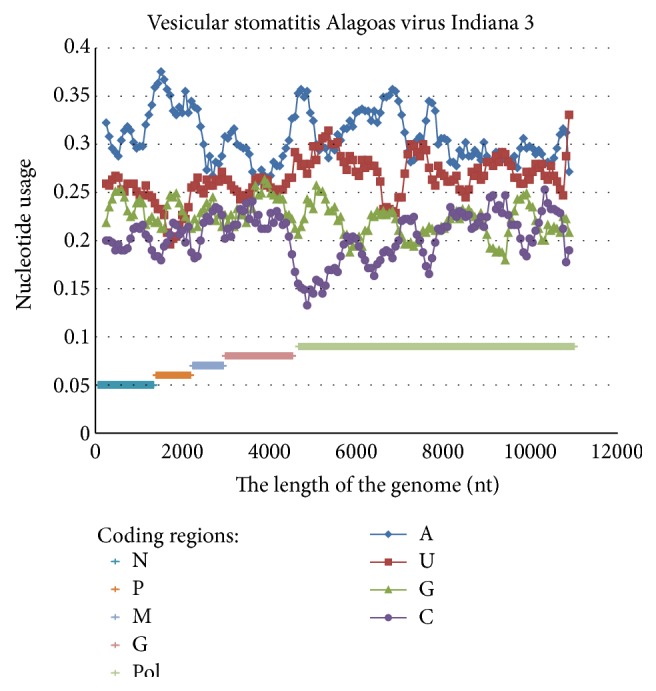
Nucleotide content distribution along the length of the Vesicular stomatitis Alagoas virus Indiana 3. Sliding window size is 490 nucleotides and the step is 70 nucleotides.

Some modern viral vectors are based on Vesicular stomatitis virus genome with inserted genes (or their parts) from Zaire ebolavirus [32]. This virus belongs to the same Mononegavirales order, as Zaire ebolavirus. As one can see in Figure 9, there is asymmetric A-pressure in the beginning of that genome and more symmetric AU-pressure in the second half. Even though this nucleotide usage distribution is similar to that from Zaire ebolavirus genome (Figure 1), there is a lack of C-rich fragments in the Vesicular stomatitis virus genome. So, the fragments of genes encoding NP part 2, VP40, and GP part 2 inserted in the given vector will accumulate numerous C to A and C to U mutations.

**Figure 10:**
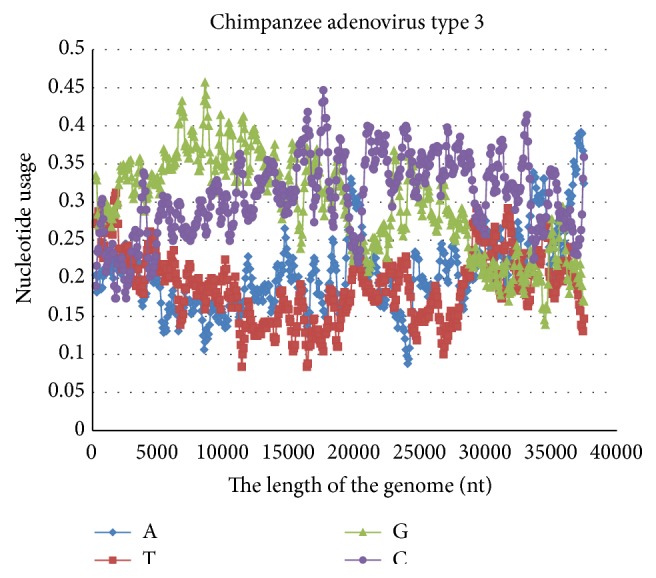
Nucleotide content distribution along the length of the Chimpanzee adenovirus type 3. Sliding window size is 490 nucleotides and the step is 70 nucleotides.

Another popular vector for Ebola vaccines creation is Chimpanzee adenovirus type 3 [33] (Figure 10). That dsDNA virus has high GC-content along the most of its genome. Unlike Zaire ebolavirus genome, Chimpanzee adenovirus type 3 genome has elevated guanine content, especially in the first half. So, there are three relatively short areas with A-pressure in the given genome (19600–20600 nt; 33500–34500 nt; 36300–37300 nt) which may be used for Zaire ebolavirus genes insertion with the aim of mimicking mutational pressure in the wild virus. Current nucleotide usage biases sometimes provide misleading information on mutational pressure directions. Because of this reason different parts of each potential viral vector should be studied with the “VVTAK VarInvar” algorithm to find the best place for entrance of Zaire ebolavirus antigens encoding regions. This work contains information about mutational pressure directions Ebola vaccine designers should look for in genomes of viruses which may be used as vectors.

## 5. Conclusions

First six genes of Marburg virus (NP, VP35, VP40, GP, VP30, and VP24) are under the asymmetric mutational A-pressure, while the last one (Pol) is under the more symmetric mutational AU-pressure.

In Zaire ebolavirus the situation with mutational biases is rather complicated. Asymmetric A-pressure can be found in NP part 1, VP35, GP part 1, GP part 3, VP30, and VP24. More symmetric AU-pressure exists in the longest Pol coding region. There is mutational AC-pressure in NP part 2, VP40, and GP part 2. This information can be used for future vaccine design studies.

## Supplementary Material

The set of unique nucleotide sequences of each *Zaire ebolavirus* coding region or its part used in this study can be found in the “DATAZE.xlsx” file. The set of unique nucleotide sequences of each *Marburg virus* coding region or its part used in this study can be found in the “DATAMV.xlsx” file.

## Figures and Tables

**Table 1:** Secondary structure of start signals of the Zaire ebolavirus reference genome according to the results of the CentroidFold algorithm.

Coding region	NP	VP35	VP40	GP	VP30	VP24	Pol
Number of nucleotide pairs in 72 nt sequence	10	9	3	15	10	9	9
Number of stems	1	1	1	1	1	2	1

**Table 2:** Amount of secondary structure (percentage of nucleotides in stems) in coding regions and their fragments for Zaire ebolavirus according to the results of the CentroidFold algorithm. Medians are given.

Coding region	NP part 1	NP part 2	VP35	VP40	GP part 1	GP part 2	GP part 3
For positive (+) strand	42,00%	33,33%	35,78%	43,22%	35,26%	34,69%	49,21%
For negative (−) strand	43,56%	43,33%	41,06%	46,28%	33,03%	49,77%	23,28%

Coding region	VP30	VP24	Pol part 1	Pol part 2	Pol part 3	Pol part 4	

For positive (+) strand	39,35%	29,76%	37,33%	37,45%	34,42%	41,21%	
For negative (−) strand	46,53%	37,17%	35,15%	42,06%	37,21%	39,85%	

**Table 3:** Amount of secondary structure (percentage of nucleotides in stems) in coding regions and their fragments for Marburg virus according to the results of the CentroidFold algorithm. Medians are given.

Coding region	NP part 1	NP part 2	VP35	VP40	GP part 1	GP part 2	GP part 3
For positive (+) strand	39,15%	35,69%	41,52%	36,80%	37,17%	20,36%	37,38%
For negative (−) strand	37,72%	39,39%	45,96%	44,53%	39,02%	39,19%	41,33%

Coding region	VP30 part 1	VP30 part 2	VP24	Pol part 1	Pol part 2	Pol part 3	Pol part 4

For positive (+) strand	6,77%	29,36%	34,79%	37,47%	37,24%	39,13%	34,31%
For negative (−) strand	23,96%	34,86%	34,00%	35,06%	33,22%	35,57%	37,34%

**Table 4:** Directions of mutational pressure in coding regions and their fragments for Zaire ebolavirus according to the “VVTAK VarInvar” algorithm results. Nucleotide usages in fourfold degenerated invariable and stable sites are compared.

Gene/fragment	NP part 1	NP part 2	VP35	VP40	GP part 1	GP part 2	GP part 3	VP30	VP24	LP
Cytosine usage										
Direction	↓	↑	↓	↑	↓	↑	↕	↓	↓	↓
Invariable	16,04%	28,18%	15,52%	27,05%	19,17%	30,16%	18,75%	17,53%	18,07%	14,22%
Stable	17,66%	27,18%	17,40%	25,10%	20,01%	28,77%	18,95%	20,37%	20,42%	16,11%

Guanine usage										
Direction	↑	↓	↓	↓	↑	↓	↓	↑	↕	↓
Invariable	16,98%	12,73%	9,48%	14,75%	19,17%	14,29%	18,75%	15,46%	22,89%	12,97%
Stable	16,58%	15,13%	11,60%	15,41%	18,33%	15,88%	19,41%	14,01%	22,89%	15,48%

Uracil usage										
Direction	↓	↓	↑	↓	↓	↓	↓	↑	↑	↑
Invariable	33,96%	23,64%	35,34%	21,31%	27,50%	22,22%	25,00%	25,77%	28,92%	33,75%
Stable	34,51%	26,24%	34,38%	26,50%	28,06%	23,54%	28,33%	25,33%	26,96%	31,46%

Adenine usage										
Direction	↑	↑	↑	↑	↑	↑	↑	↑	↑	↑
Invariable	33,02%	35,45%	39,66%	36,89%	34,17%	33,33%	37,50%	41,24%	30,12%	39,06%
Stable	31,24%	31,45%	36,63%	32,99%	33,60%	31,82%	33,31%	40,29%	29,73%	36,95%

**Table 5:** Directions of mutational pressure in coding regions and their fragments for Zaire ebolavirus according to the “VVTAK VarInvar” algorithm results. Nucleotide usages in invariable and stable twofold degenerated sites from third codon positions are compared.

Gene/fragment	NP part 1	NP part 2	VP35	VP40	GP part 1	GP part 2	GP part 3	VP30	VP24	LP
Cytosine usage										
Direction	↑	↓	↑	↑	↑	↓	↕	↑	↑	↓
Invariable	19,80%	25,32%	20,87%	25,56%	31,48%	37,50%	27,91%	14,41%	25,29%	18,08%
Stable	18,75%	27,87%	19,29%	23,97%	31,22%	39,64%	27,79%	14,02%	23,97%	19,34%

Guanine usage										
Direction	↓	↑	↓	↓	↓	↑	↓	↓	↓	↓
Invariable	24,75%	23,38%	22,61%	26,67%	18,52%	12,50%	18,60%	21,62%	18,39%	16,67%
Stable	25,69%	21,87%	24,87%	27,74%	21,36%	11,82%	20,34%	22,01%	19,16%	17,31%

Uracil usage										
Direction	↓	↓	↑	↑	↑	↓	↑	↑	↓	↑
Invariable	27,72%	26,62%	26,96%	32,22%	24,07%	21,43%	32,56%	28,83%	24,14%	35,92%
Stable	28,26%	27,30%	25,75%	29,88%	23,46%	22,13%	28,21%	26,29%	26,03%	34,65%

Adenine usage										
Direction	↑	↑	↑	↓	↑	↑	↓	↓	↑	↑
Invariable	27,72%	24,68%	29,57%	15,56%	25,93%	28,57%	20,93%	35,14%	32,18%	29,34%
Stable	27,30%	22,96%	30,09%	18,41%	23,95%	26,42%	23,66%	37,68%	30,84%	28,69%

**Table 6:** Directions of mutational pressure in coding regions and their fragments for Marburg virus according to the “VVTAK VarInvar” algorithm results. Nucleotide usages in fourfold degenerated invariable and stable sites are compared.

Gene/fragment	NP	VP35	VP40	GP part 1	GP part 2	GP part 3	VP30 part 1	VP30 part 2	VP24	LP
Cytosine usage										
Direction	↓	↓	↓	↓	↓	↓	↓	↓	↓	↓
Invariable	11,40%	15,69%	20,83%	16,22%	20,00%	17,65%	16,67%	8,70%	6,25%	5,22%
Stable	17,07%	23,06%	22,19%	25,65%	34,25%	25,15%	25,45%	18,02%	16,69%	14,19%

Guanine usage										
Direction	↓	↓	↓	↓	↓	↕	↓	↓	↓	↓
Invariable	14,04%	3,92%	14,58%	8,11%	0,00%	5,88%	0,00%	0,00%	6,25%	6,72%
Stable	15,29%	10,19%	18,57%	14,80%	8,00%	6,23%	0,89%	15,97%	14,15%	12,13%

Uracil usage										
Direction	↓	↓	↕	↑	↓	↓	↓	↑	↑	↑
Invariable	32,46%	23,53%	31,25%	35,14%	10,00%	35,29%	25,00%	43,48%	40,63%	45,15%
Stable	33,36%	31,30%	30,77%	31,35%	24,43%	38,39%	36,46%	30,91%	34,40%	38,05%

Adenine usage										
Direction	↑	↑	↑	↑	↑	↑	↑	↑	↑	↑
Invariable	42,11%	56,86%	33,33%	40,54%	70,00%	41,18%	58,33%	47,83%	46,88%	42,91%
Stable	34,27%	35,44%	28,46%	28,20%	33,31%	30,23%	37,20%	35,10%	34,76%	35,63%

**Table 7:** Directions of mutational pressure in coding regions and their fragments for Marburg virus according to the “VVTAK VarInvar” algorithm results. Nucleotide usages in invariable and stable twofold degenerated sites from third codon positions are compared.

Gene/fragment	NP	VP35	VP40	GP part 1	GP part 2	GP part 3	VP30 part 1	VP30 part 2	VP24	LP
Cytosine usage										
Direction	↓	↓	↓	↓	↓	↓	↑	↑	↓	↓
Invariable	12,23%	18,52%	18,87%	13,89%	33,33%	2,33%	12,50%	20,78%	12,77%	9,82%
Stable	17,30%	21,57%	26,10%	24,29%	34,09%	13,82%	4,76%	18,25%	21,64%	16,44%

Guanine usage										
Direction	↓	↓	↓	↓	↓	↕	↓	↕	↕	↓
Invariable	14,39%	16,67%	16,98%	13,89%	11,11%	18,60%	0,00%	19,05%	23,40%	9,60%
Stable	21,01%	18,77%	17,86%	15,89%	14,89%	18,39%	6,72%	18,99%	22,89%	15,15%

Uracil usage										
Direction	↑	↓	↓	↑	↑	↑	↓	↑	↓	↑
Invariable	38,13%	25,93%	32,08%	33,33%	22,22%	46,51%	25,00%	35,71%	25,53%	46,21%
Stable	33,03%	26,73%	33,74%	31,96%	18,85%	37,27%	34,22%	31,22%	26,40%	38,48%

Adenine usage										
Direction	↑	↑	↑	↑	↑	↑	↑	↑	↑	↑
Invariable	35,25%	38,89%	32,08%	38,89%	33,33%	32,56%	62,50%	40,48%	38,30%	34,38%
Stable	28,66%	32,92%	22,30%	27,86%	32,17%	30,52%	38,28%	31,55%	29,07%	29,92%
